# Clinical outcomes of posterior fossa arteriovenous malformations: a single center experience

**DOI:** 10.1007/s00701-024-06116-9

**Published:** 2024-05-14

**Authors:** Ioana Miron, Viorel M. Prună, Dan Visarion, Andrei Giovani, Aurelia M. Sandu, Felix M. Brehar, George E. D. Petrescu, Radu M. Gorgan

**Affiliations:** 1 Department of Neurosurgery, Carol Davila University of Medicine and Pharmacy, Bucharest, Romania; 2 Department of Neurosurgery, Bagdasar-Arseni Clinical Emergency Hospital, Bucharest, Romania

**Keywords:** Posterior fossa arteriovenous malformations, Prognostic factors, Clinical outcome, Treatment modalities

## Abstract

**Background:**

Posterior fossa arterio-venous malformations (pfAVMs) are challenging lesions due to the anatomical particularities of the posterior fossa, and the high incidence of hemorrhagic presentation. The two most important goals when treating AVMs are preserving neurological function and preventing rupture, or a second hemorrhage. The aim of this study was to analyze the clinical and imaging features of pfAVMs to identify the factors that influence the prognosis of these patients.

**Methods:**

We conducted a single-center retrospective observational study that included patients treated at our institution with pfAVMs between January 1997 and December 2021.

**Results:**

A total of 48 patients were included. A good modified Rankin score (mRS) was observed in 33 cases (69%) at presentation. Thirty-four patients (71%) presented with a ruptured AVM. Out of these, 19 patients (40%) had intraventricular hemorrhage. Microsurgical resection was performed in 33 cases (69%), while in the other cases, the patients opted for conservative management (7 cases, 15%), stereotactic radiosurgery (SRS) (6 cases, 12%), or endovascular treatment (2 cases, 4%). Patients ≤ 30 years old were more prone to hemorrhagic presentation (OR: 5.23; 95% CI: 1.42–17.19; *p* = 0.024) and this remained an independent risk factor for rupture after multivariate analysis as well (OR: 4.81; 95% CI: 1.07–21.53; *p* = 0.040). Following multivariate analysis, the only factor independently associated with poor prognosis in the surgically treated subgroup was a poor clinical status (mRS 3–5) at admission (OR: 96.14; 95% CI: 5.15–1793.9; *p* = 0.002).

**Conclusions:**

Management of posterior fossa AVMs is challenging, and patients who present with ruptured AVMs often have a poor clinical status at admission leading to a poor prognosis. Therefore, proper and timely management of these patients is essential.

## Introduction

Arteriovenous malformations located in the posterior fossa (pfAVMs) are rare lesions, comprising between 7 and 15% of all AVMs [19]. Due to the anatomical particularities of the posterior fossa (a confined space with many vital structures, i.e., brainstem, fourth ventricle, deep cerebellar nuclei), any hemorrhage in this location may have hazardous consequences. For this reason, ruptured pfAVMs have a fatality rate between 10 and 30% [17], or even higher than 50% in other series [7]. In comparison with the AVMs located in the supratentorial compartment, the infratentorial ones present more often with rupture, estimated between 75 and 92% in some studies [1, 29]. This may also be explained by the fact that patients with supratentorial AVMs, even though most commonly present with hemorrhage, can also manifest seizures, which can lead to an earlier diagnosis, before rupture [4, 24]. Khaw et al. and Rodriguez-Hernandez et al. reported that the percentage of supratentorial arterio-venous malformations that manifest with seizures represents the difference between posterior fossa AVMs and supratentorial AVMs diagnosed with rupture [12, 20]. Hence, the fact that pfAVMs do not present with epileptic seizures might be the explanation for a much higher rate of hemorrhaging presentation in comparison with their supratentorial counterparts. Given these particularities and their aggressive natural history, pfAVMs yield significant challenges. The two most important goals when treating AVMs are preserving neurological function and preventing rupture, or a second hemorrhage. Choosing the optimal therapeutic approach in order to achieve these goals is a complex process that needs to take into account the clinical status of the patient and the characteristics of the AVM. Existing literature on pfAVMs predominantly comprises descriptive studies. While these contributions are invaluable, there remains a critical need for a detailed analysis of risk factors for hemorrhagic presentation and prognostic factors. Therefore, the aim of this study was to analyze the clinical and imaging features from a large retrospective series of pfAVMs, in order to identify the factors that influence the prognosis of these patients.

## Materials & methods

### Patient population

We conducted a single-center, retrospective observational study that included patients with posterior fossa AVMs who were admitted at our institution between January 1997 and December 2021. Included patients had a pfAVM diagnosis determined by digital subtraction angiography (DSA), CT angiography (CTA), MR angiography (MRA) investigations or confirmed by histological findings. Patients with AVMs located in both the supratentorial and infratentorial compartments were excluded. Also, patients treated before at other institutions, or who presented in a very poor clinical condition (i.e., Glasgow Coma Scale – GCS 3 points and non-reactive pupils) that did not tolerate further angiographic studies were not included in the present study.

### Data management

We collected patient demographic, clinical, and imaging data, including: age, gender, GCS, modified Rankin scale (mRS) at admission, presenting symptoms, any neurologic deficits, grading on Spetzler-Martin (SM) and Lawton-Young supplementary scale, presence of hemorrhage, including intraventricular hemorrhage (IVH), radiological characteristics (location, eloquence, venous drainage pattern, associated aneurysms, arterial feeders), treatment modality, complications, clinical outcome using mRS at discharge, and follow-up data.

Regarding location, pfAVMs were divided into the following groups: cerebellum (cerebellar hemisphere or vermis), cerebello-pontine angle (CPA), brainstem or complex (multiple territories). The pattern of venous drainage was defined as superficial or deep. The cases that included both superficial and deep venous drainage were dichotomized as deep. A good clinical outcome was defined as mRS < 3. The number of arterial feeders was also dichotomized, the categories being ≤ 2 or > 2 feeders.

The rupture status was noted in a binary manner, while the presence of IVH was also separately recorded for each case. Aneurysms were categorized as intranidal, flow-related or unrelated to the AVM.

The cases that had missing values for a variable that was analyzed, were excluded from that specific analysis.

Treatment modalities included conservative (“wait and see”), surgical resection, endovascular embolization, stereotactic radiosurgery (SRS), or a combination of the aforementioned methods.

### Statistical analysis

Statistical analysis was performed using IBM SPSS Statistics for Windows, version 29 (IBM Corp., Armonk, N.Y., USA) and GraphPad Prism version 9.5.1 for Windows (GraphPad Software, San Diego, California USA). Fisher’s exact test was used in univariate analysis of the categorical variables. Odds ratios and their confidence intervals were determined using the Baptista-Pike method or Woolf method as appropriate for the analysis. When performing the multivariate analysis, due to the relatively small cohort, depending on the analysis we have used appropriate methods for variable selection as described here [9].We used the log-rank test to analyze rupture-free survival. Statistical significance was considered at *p* < 0.05.

The present retrospective study was approved by the hospital’s ethics committee.

## Results

Between January 1997 and December 2021, 53 patients with posterior fossa AVMs were admitted at our institution, out of which 48 met the inclusion criteria. The median age was 27.5 years (range, 9–73 years) and 26 patients (54%) were female. Patient characteristics are summarized in Table 1.

**Table 1:** Characteristics of patients with posterior fossa AVMs

Characteristic	Number (%)
Gender	
Female	26 (54%)
Male	22 (46%)
Age (years)	
Range	9–73
Median age	27.5
Clinical condition	
GCS ≥ 14	35 (73%)
GCS 13–9	9 (19%)
GCS ≤ 8	4 (8%)
mRS at presentation	
mRS 1–2	33 (69%)
mRS 3–5	15 (31%)
Rupture status	
Ruptured	34 (71%)
Unruptured	14 (29%)
IVH	19 (40%)
Neurologic Deficits	
Cranial nerve deficits	21 (44%)
Other focal neurological deficits	7 (15%)
Location	
Cerebellum	40 (83%)
Brainstem	5 (11%)
CPA	2 (4%)
Complex	1 (2%)

*CPA - *cerebello−pontine angle, *GCS - *glasgow coma score, *IVH - *intraventricular hemorrhage, *mRS - *modified Rankin scale

### Clinical presentation

On admission, 35 patients (73%) presented in a good clinical status (GCS ≥ 14), while in 4 cases (8%) the patients were unconscious (GCS ≤ 8). A good mRS score (mRS 1–2) was observed in 33 cases (69%) at presentation. Thirty-four patients (71%) presented with a ruptured AVM. Out of these, 19 patients (40%) had intraventricular hemorrhage. Twenty-one patients (44%) showed cranial nerve deficits on admission, while seven patients (15%) presented other focal neurological deficits.

### Characteristics of AVMs

The AVMs were located in the cerebellum in 40 cases (83%) and in 5 cases (11%) in the brainstem. In two patients (4%) the AVMs were found in the cerebello-pontine angle (CPA) and in one case (2%) the AVM occupied the right cerebellar hemisphere, CPA, and brainstem.

Preoperative DSA was available in 43 cases (90%), while in 4 cases (8%) MRA was performed and in one case (2%) of a young patient with a ruptured AVM, only a CTA was available.

**Fig. 1:**
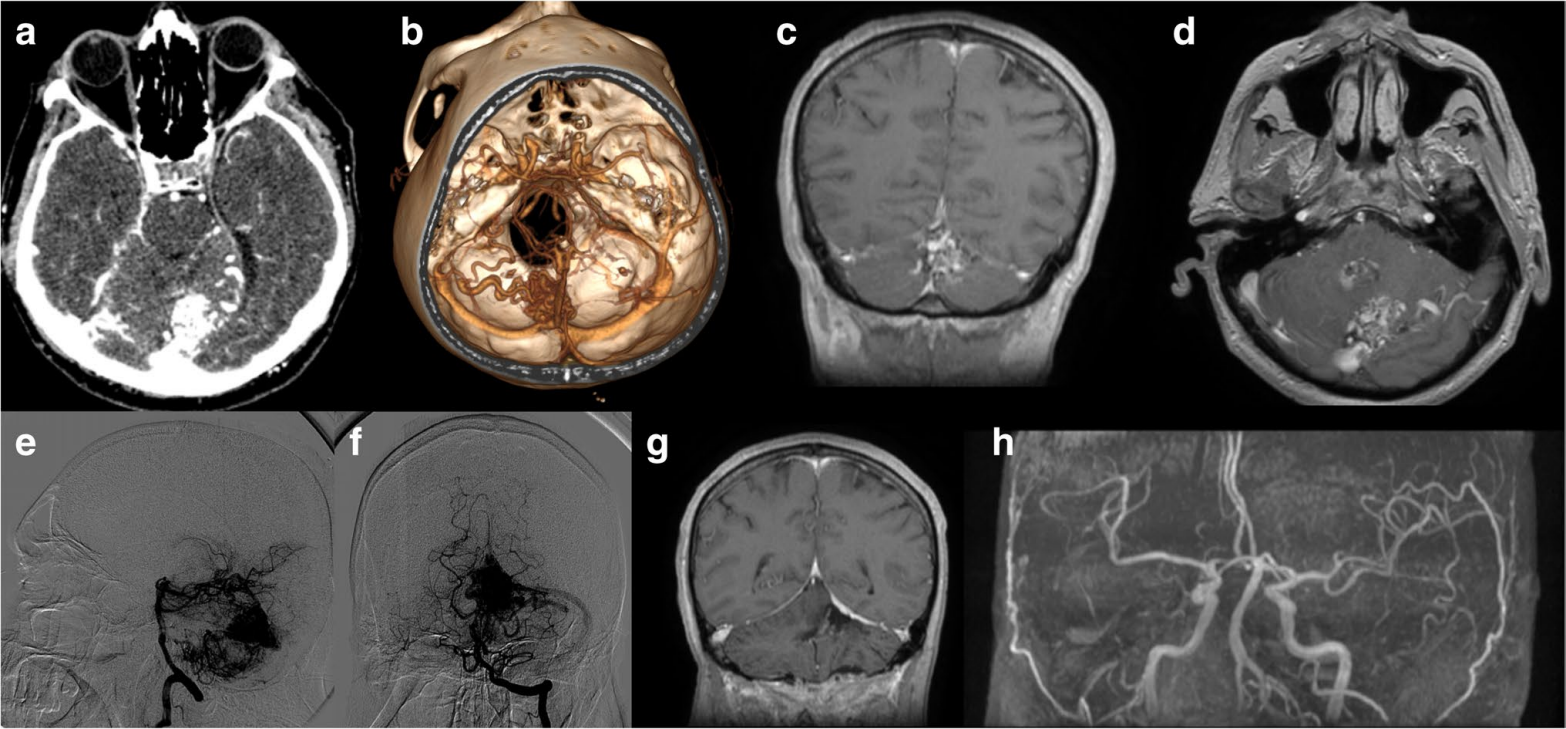
Illustrates the case of 56-year-old male that presented with headache and ataxia; imaging studies (**a**, **b** - CT scan with CTA; **c**, **d** – MRI; **e**, **f** – DSA) showed an unruptured vermian AVM with feeders from PICA and left cerebellar artery; **g**, **f** – postoperative MRI and MRA showed complete resection of the AVM

Most of the AVMs were SM grade II (27 cases, 56%), while 8 AVMs (17%) were SM grade III, 7 (15%) were SM grade IV, and 6 AVMs (12%) were SM grade I. The median SM grade was 2 for the cerebellar AVMs, and 3 for the brainstem AVMs. The two CPA AVMs were SM grade II, and the SM grade for the complex (cerebellar, brainstem and CPA) AVM was IV (Table 2). Five patients (11%) had associated aneurysms, in three cases being intranidal, while in two were flow related aneurysms. Deep venous drainage was observed in thirty-seven cases (77%). Figure 1 illustrates an unruptured pfAVM.

**Table 2:** Radiologic characteristics of 48 patients with posterior fossa AVMs

Characteristic	Number (%)
Spetzler-Martin (SM) scale	
SM I	6 (12%)
SM II	27 (56%)
SM III	8 (17%)
SM IV	7 (15%)
Median SM grade	
Cerebellar	2
Brainstem	3
Supp-SM grade	
Supp-SM 2	1 (2%)
Supp-SM 3	9 (19%)
Supp-SM 4	11 (23%)
Supp-SM 5	14 (29%)
Supp-SM 6	6 (13%)
Supp-SM 7	3 (6%)
Supp-SM 8	3 (6%)
Supp-SM 9	1 (2%)
Associated aneurysms	5 (11%)
Intranidal	3 (6%)
Flow related	2 (5%)
Deep venous drainage	37 (77%)
Arterial feeders	
1–2 arterial feeders	23 (48%)
≥ 3 arterial feeders	21 (44%)
undetermined	4 (8%)

Supp-SM - Supplementary Spetzler-Martin grade (Spetzler-Martin grade+Lawton-Young grade)

We observed a statistically significant difference between the characteristics of the AVMs based on the SM scale and the clinical status based on the GCS scale (*p* = 0.018). Therefore, 84.4% patients with SM I-II AVMs had a good clinical status at presentation (GCS 14–15), while only 50% of patients with SM III-IV AVMs had a similar status. Moreover, there was no significant association between SM I-II and SM III-IV AVMs regarding rupture status (*p* = 0.178).

### Factors associated with hemorrhagic presentation

**Fig. 2:**
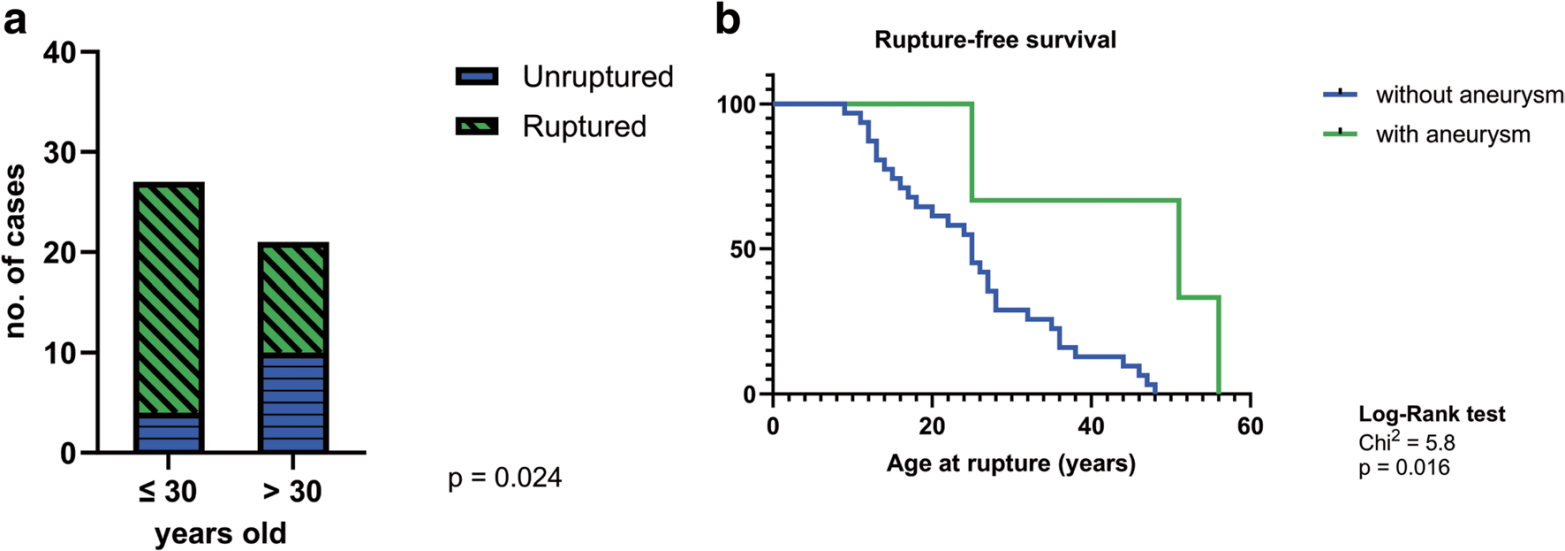
Factors influencing the hemorrhagic presentation of patients with pfAVMs; **a** – patients ≤ 30 years old are more prone to present with a ruptured pfAVM; **b** – pfAVMs with associated aneurysms tend to rupture later than pfAVMs without associated aneurysms (HR: 0.32; 95% CI: 0.14–0.71; *p* = 0.016)

Table 3 illustrates the results of the univariate and multivariate analyses of factors associated with ruptured pfAVMs. Following univariate analysis, younger patients, ≤ 30 years old were more prone to hemorrhagic presentation (OR: 5.23; 95% CI: 1.42–17.19; *p* = 0.024) and this remained an independent risk factor for rupture after multivariate analysis as well (OR: 4.81; 95% CI: 1.07–21.53; *p* = 0.040) (Fig. 2a). While it marginally missed reaching statistical significance, there seems to be an association between superficial location of pfAMVs and rupture in our cohort (OR: 4.35; 95% CI: 1.17–18.71; *p* = 0.058). Although male patients were more likely to present with ruptured AVMs, this finding did not reach statistical significance in univariate analysis (OR: 4.64; 95% CI: 1.18–17.22; *p* = 0.054). However, upon inclusion of gender in the multivariate analysis, there was a statistically significant difference (OR: 5.21; 95% CI: 1.01–26.77; *p* = 0.048). We did not find any association between other factors such as the presence of a single feeding artery, deep venous drainage or a nidus size > 3 cm, and hemorrhagic presentation. It is important to highlight that the presence of an associated aneurysm (while not statistically significant) might be associated with a lower rupture rate (OR: 0.58; 95% CI: 0.11–3.62; *p* = 0.621) and therefore we compared the time to rupture (measured by age at diagnosis) in patients with associated aneurysms and those without (Fig. 2b). When applying the log-rank test, there was a statistically significant difference between the time to rupture between the two groups, patients with aneurysms having a median time to rupture of 51 years, and those without of 21 years (HR: 0.32; 95% CI: 0.14–0.71; *p* = 0.016).

**Table 3:** Risk factors associated with hemorrhagic presentation

Variable	Univariate analysis		Multivariate analysis	
	OR (95% CI)	*p* value	OR (95% CI)	*p* value
Age ≤ 30 years old	5.23 (1.42–17.19)	0.024^*^	4.81 (1.07–21.53)	0.040^*^
Superficial location	4.35 (1.17–18.71)	0.058	5.22 (0.96–28.43)	0.056
Male	4.64 (1.18–17.22)	0.054	5.21 (1.01–26.77)	0.048^*^
Single feeding artery	1.53 (0.35–5.93)	0.727	NT	NT
Deep venous drainage	1.53 (0.42–5.67)	0.708	NT	NT
Associated aneurysm	0.58 (0.11–3.62)	0.621	NT	NT
Nidus size > 3 cm	1.80 (0.53–6.74)	0.503	NT	NT

* - statistical significance; *NT* - not tested

### Treatment

Forty-three patients (90%) received single-modality treatment, whereas multimodality treatment (any combination of radiosurgery, embolization, and surgery) was performed in 5 cases (10%).

Microsurgical resection was performed in 33 cases (69%), while in the other cases, the patients opted for conservative management (7 cases, 15%), stereotactic radiosurgery (SRS) (6 cases, 12%), or endovascular treatment (2 cases, 4%). The median time to surgery for cases that presented with rupture was 2 days (range, 0–40 days). However, for cases that presented in poor clinical status, GCS < 8, (*n* = 4) the median time to surgery was shorter, at 0.5 days (range, 0–4 days). Following surgical treatment, complete resection of the AVMs was achieved in 25 cases (76%). The six cases treated with radiosurgery were SM grade I or II, having less than 3 cm in size. Of the five cases that received multimodality treatment, three had SRS following surgical resection and one had both embolization and SRS after surgery. In one case, SRS was used to treat the AVM and coils embolization for the ruptured PICA flow-related aneurysm.

### Conservative management

Of the seven cases that were managed conservatively, one patient presented with re-rupture during the follow-up period, at 61 months after the initial presentation, in a poor clinical status and was treated surgically. Another patient, with a brainstem AVM, presented with a re-rupture and secondary hydrocephalus for which a ventriculoperitoneal shunt was placed. The patient refused surgery for the AVM.

The median mRS at presentation for this subgroup was 2 (range, 1–2), while at discharge the median mRS was 1 (range, 0–1). The median follow-up time for this group was 3 months (range, 1–156 months). Two patients were lost during follow-up. Median mRS at the last follow-up for patients treated conservatively was 3 (range, 0–5).

### Clinical Outcome

**Fig. 3:**
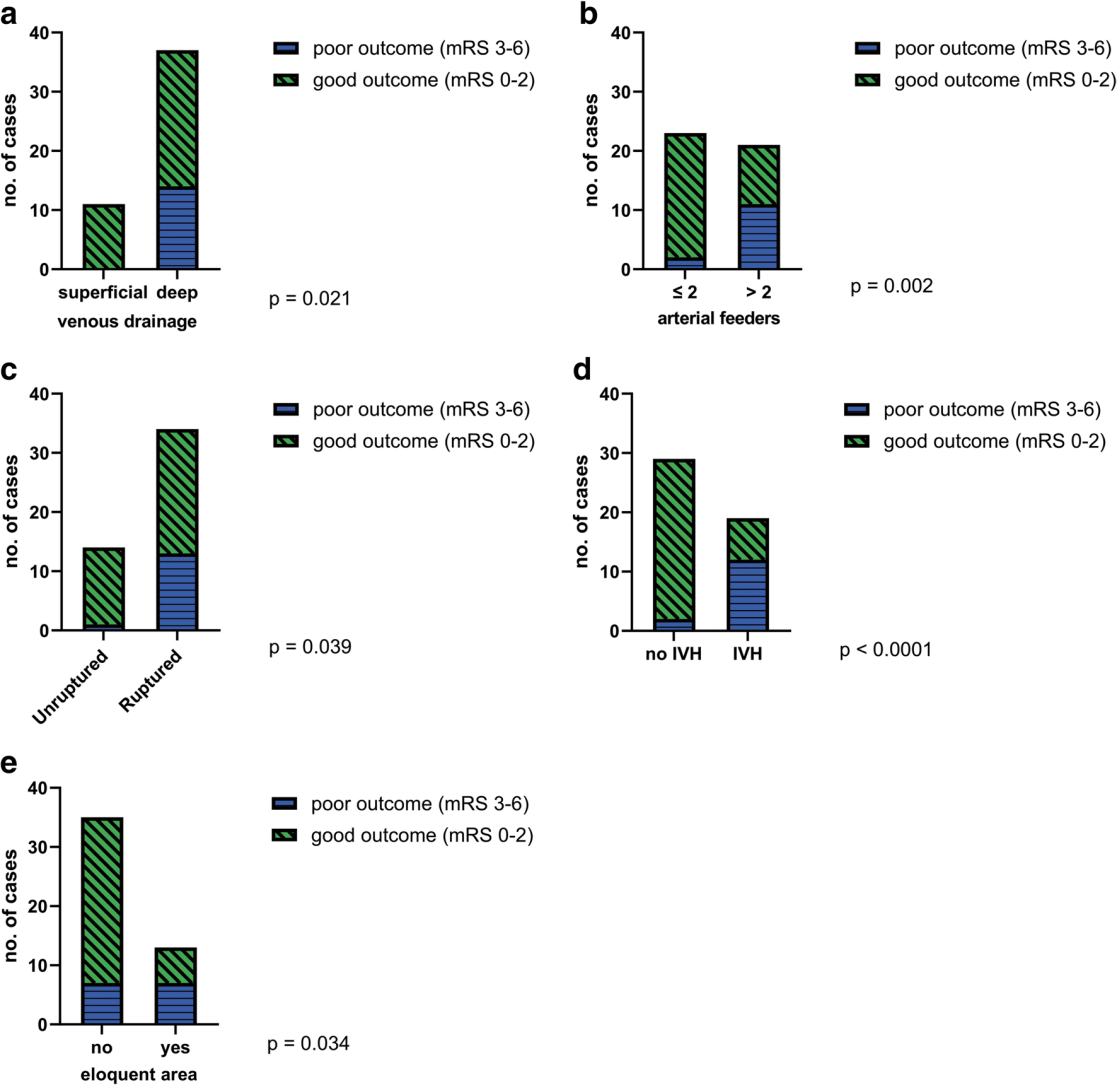
Factors influencing the outcome of patients with posterior fossa AVMs based on the modified Rankin scale (mRS). *p* values are shown; **a** – deep venous drainage is associated with poor outcome; **b** – the presence of more than 2 arterial feeders is a predictor for poor outcome; **c** – ruptured posterior fossa AVMs have a poorer outcome compared to unruptured ones; **d** – the presence of intraventricular hemorrhage (IVH) has a negative prognostic value; **e**—the location of the AVM in an eloquent area is associated with a poor outcome

Deep venous drainage was significantly associated with poor outcome (*p* = 0.021). Of the 37 AVMs that had deep venous drainage, 14 (38%) had a poor outcome (mRS 3–6), while all the AVMs from the superficial drainage group, represented by 11 cases, had a good outcome (mRS 0–2) (Fig. 3a).

Moreover, we observed a statistically significant correlation between the number of arterial feeders and the patient’s outcome (*p* = 0.002). Patients with AVMs that had only one or two arterial feeders had a much better outcome (91%) than patients with AVMs with more than two feeding arteries (48%) (Fig. 3b).

Regarding the bleeding status of the lesion, unruptured AVMs had an overall better outcome in comparison with the ruptured ones (*p* = 0.039), with 93% of unruptured cases having a mRS ≤ 2 at discharge (Fig. 3c). Of the 34 patients that presented with hemorrhage, 21 (62%) had a good clinical and neurological state at discharge. Univariate analysis revealed that intraventricular hemorrhage (IVH) was statistically associated with a negative prognosis (*p* < 0.001), 63% of these patients having a poor outcome, while only 7% of patients without IVH had a poor mRS at discharge (mRS 3–6) (Fig. 3d). Patients aged ≤ 30 years old presented a statistically significant higher rupture rate (85%) than patients older than 30 years old (52%) (*p* = 0.024). After analyzing the subgroup of patients that presented with ruptured AVMs, intraventricular hemorrhage remained a strong negative prognostic factor (*p* = 0.001). Most patients (93%) with ruptured AVMs that did not have IVH had a good outcome, while 63% of those with IVH had a poor outcome. Another factor that influenced the outcome in a statistically significant manner was the location of the AVM in an eloquent area. Patients with a non-eloquent AVM had a good outcome in 80% of the cases, while patients with eloquent AVMs had a good outcome only in 46% of the cases (*p* = 0.034) (Fig. 3e).

When comparing the subgroup of unruptured pfAVMs (*n* = 14), there was no statistically significant difference regarding the outcome between the patients that underwent surgical resection (*n* = 10) and the ones that were managed through endovascular, radiosurgery or conservative (*p* = 0.714).

**Fig. 4:**
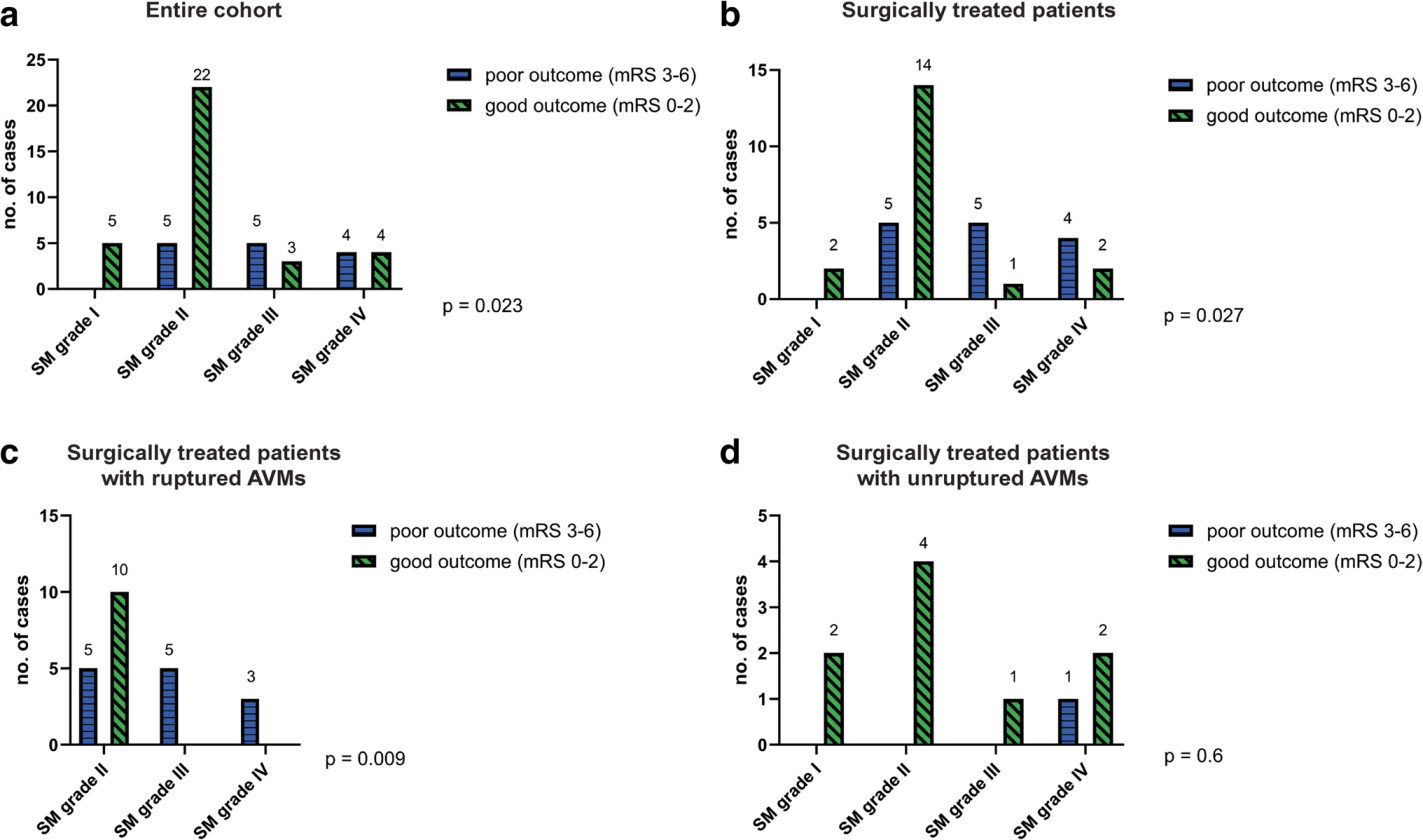
The impact of the Spetzler-Martin scale on the outcome of patients with posterior fossa AVMs. *p* values are shown; **a** – across the entire cohort, patients with SM grade I or II AVMs had a better outcome than patients with SM grade III or IV AVMs; **b** – impact of SM scale on the outcome of the 33 patients that underwent surgical resection; **c** – SM grade correlates with the outcome in patient with ruptured AVMs that underwent surgical treatment; **d** – in the subgroup of patients with unruptured AVMs that underwent surgery, SM scale does not influence the outcome in our series, since the majority of patients from this subgroup had a good outcome following surgery, regardless of their SM grade

Fisher’s exact test showed that the Spetzler-Martin scale influences the outcome of patients with pfAVMs, patients with SM grade I or II having a better outcome than patients with SM grade III or IV malformations (*p* = 0.023) (Fig. 4a). This also applies for surgically treated patients (Fig. 4b) and especially in the case of ruptured AVMs that were surgically treated (Fig. 4c). However, in the case of unruptured AVMs that were surgically treated, the SM grade does not influence the outcome in our cohort, since the majority of the patients from this subgroup had a favorable outcome, regardless of the SM grade (Fig. 4d).

### Outcome of surgically treated patients

**Fig.5:**
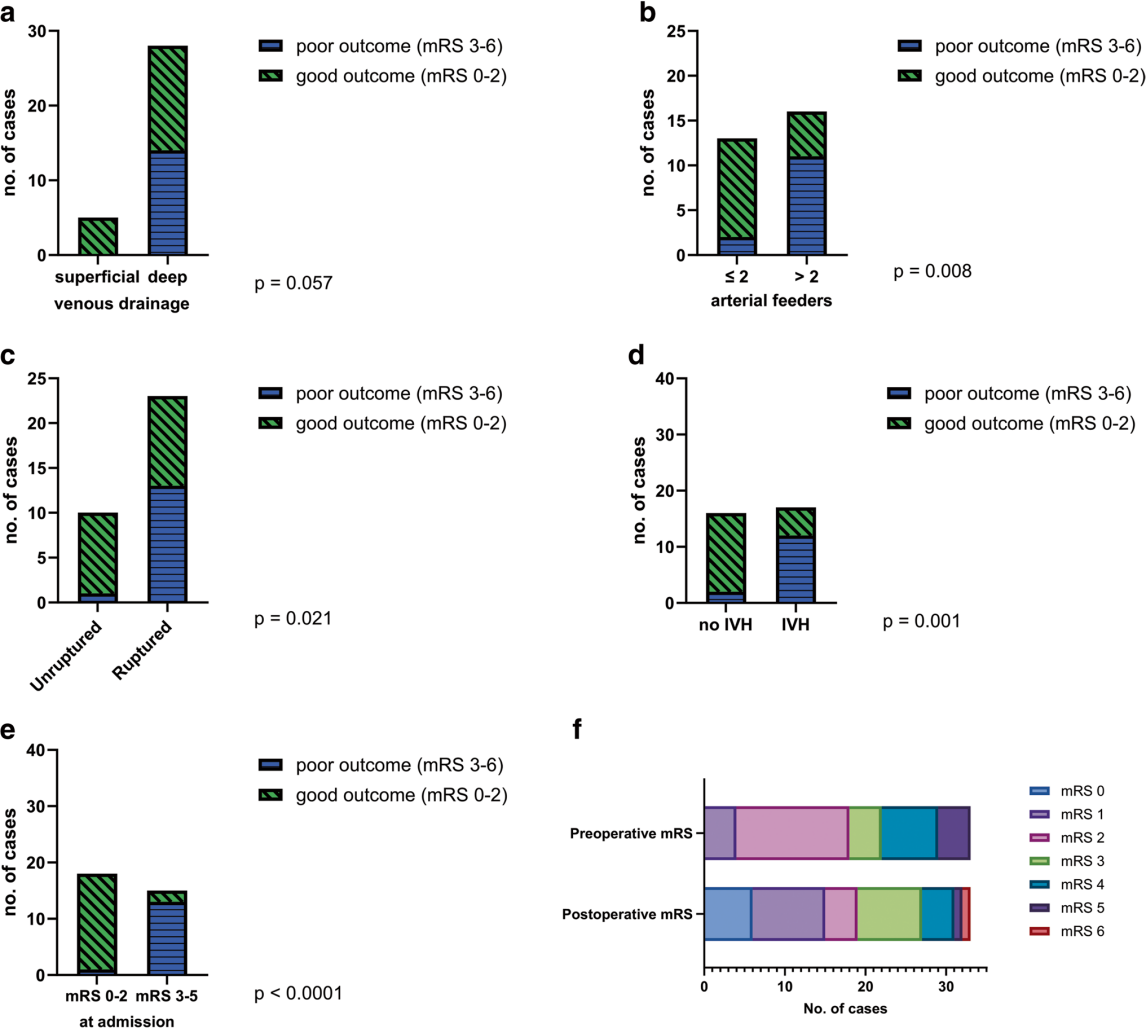
Factors influencing the outcome of patients that underwent surgical resection for a posterior fossa AVM, based on the modified Rankin scale (mRS). *p* values are shown; **a** – while not statistically significant, deep venous drainage is associated with poor outcome; **b** – the presence of more than 2 arterial feeders is a predictor for poor outcome; **c** – following surgery, ruptured posterior fossa AVMs have a poorer outcome compared to unruptured ones; **d** – the presence of intraventricular hemorrhage (IVH) at admission negatively affects the outcome; **e** – poor mRS (3–5) at admission influences the outcome; **f** – distribution of mRS score at admission and following surgery

We analyzed the subgroup of patients that underwent surgical resection (*n* = 33) to determine the factors that affect the postoperative outcome (Fig. 5). While not statistically significant, the type of venous drainage influenced the postoperative results, in a similar manner as mentioned before: 50% of the cases with deep venous drainage had a poor outcome, while 100% of those with only superficial drainage had a good outcome, with a mRS ≤ 2 (*p* = 0.057). A strong statistical correlation was demonstrated between the number of arterial feeders and the clinical and neurological results following surgery (*p* = 0.008). Thus, 85% of the patients whose AVMs had ≤ 2 feeders had a favorable outcome (mRS 0–2), while 69% of those with 3 or more feeders had a poor mRS (mRS 3–6) after treatment.

Rupture status had a statistically significant impact on the postoperative outcome (*p* = 0.021). Of all unruptured posterior fossa AVMs that were operated in our clinic, 90% had a good outcome after surgery. As mentioned before, 34 patients presented with a ruptured AVM and 23 of them were operated. After the surgery, only 10 (44%) had a good outcome (mRS 0–2). Similarly, intraventricular hemorrhage at presentation was associated with a negative prognosis, 70% of these patients having a mRS ≥ 3 at discharge (*p* = 0.001).

Moreover, the clinical status at admission defined as the mRS score was significantly associated with the postoperative outcome, 94% of the patients with good clinical status at presentation having a good outcome (Fig. 5e and f).

After including the variables in the multivariate analysis, the only factor independently associated with poor prognosis in the surgically treated patients was a poor clinical status (mRS 3–5) at admission (OR: 96.14; 95% CI: 5.15–1793.9; *p* = 0.002) (Table 4).

**Table 4:** Factors associated with poor outcome in surgically treated patients

Variable	Univariate analysis		Multivariate analysis	
	OR (95% CI)	*p* value	OR (95% CI)	*p* value
Poor mRS (3–5) at admission	110.5 (9.12 – 1208)	< 0.0001^*^	96.14 (5.15 – 1793.9)	0.002^*^
> 2 arterial feeders	12.1 (2.09 – 63.79)	0.008^*^	5.39 (0.26 – 111.98)	0.276
IVH	16.8 (2.87 – 86.02)	0.001^*^	13.26 (0.57 – 307.44)	0.107
Ruptured AVM	11.7 (1.55 – 136.6)	0.021^*^	NT	NT
Deep venous drainage	11.0 (0.55 – 217.7)	0.057	NT	NT

* - statistical significance; *AVM - *arteriovenous malformation; *IVH - *intraventricular hemorrhage; *mRS - *modified Rankin Scale; *NT - *not tested

Seven patients (21%) presented hydrocephalus. IVH was statistically significant associated with the occurrence of hydrocephalus (*p* = 0.007), since all patients with hydrocephalus presented IVH at admission.

### Complications

One patient (3%), that had hydrocephalus for which a ventriculoperitoneal shunt was placed, presented with meningitis after approximately seven years since the surgical resection of the AVM. Two patients (6%) developed a hematoma in the surgical resection cavity and needed reintervention to evacuate the hematoma. Two patients (6%) had postoperative CSF fistulae that were resolved with the placement of external lumbar drainages. One elderly patient (3%) with multiple comorbidities who underwent surgery for a grade IV AVM was admitted to the intensive care unit and succumbed two months later.

### Outcome at follow-up

Median follow-up period was 24 months (range, 1–264 months). There was a statistically significant association between mRS at discharge and mRS at the last follow-up visit (*p* < 0.001). Among the subgroup of patients with good mRS score (mRS 0–2) at discharge, 28 out of 32 (87.5%) maintained a good outcome at their last follow-up visit. Conversely, only 3 out of 13 patients (23.1%) with poor mRS score (mRS 3–6) at discharge had a good mRS score at follow-up.

## Discussions

Infratentorial AVMs constitute a heterogenous group regarding prognostic and treatment considerations and data available in the literature is mostly limited to descriptive studies. By analyzing a relatively large cohort of pfAVMs, we critically reviewed the impact of clinical and radiological factors on hemorrhagic presentation, as well as the factors that influence the outcome of these patients, with a focus on the surgical treatment.

### Clinical interpretation of factors influencing AVM rupture

In our series, thirty-four patients (71%) presented with a ruptured AVM, a finding consistent with the series reported by Stein et al. [25] and Yang et al. [29]. Although there was a high rate of hemorrhagic presentation in our series (71%), the majority of the patients presented in good clinical status based on the GCS (GCS ≥ 14 in 73% of the cases). This can be explained by the relatively high number of AVMs SM grade I and II. Moreover, other series suggest similar rates. For example, Roberto et al. showed an 82.6% rupture rate and 78.2% of the patients were GCS 14 or 15.

Patient’s age might be a risk factor for hemorrhagic presentation, but data on this subject are very conflicting. In our cohort, patients ≤ 30 years-old presented a much higher rupture rate (85%), in comparison with those older than 30 years old (52%, *p* = 0.024). This remained an independent factor associated with hemorrhagic presentation after multivariate analysis as well (OR: 4.81; 95% CI: 1.07–21.53; *p* = 0.040). Similar results were reported by Tong et al., young age at presentation being an independent risk factor for rupture, in univariate and multivariate analysis [28]. On the contrary, other studies demonstrated positive association between increasing age (> 60 years-old) and hemorrhagic presentation [22, 23]. While in univariate analysis, there was not a statistically significant difference between the male and female patients regarding hemorrhagic presentation, male patients were more prone to present with rupture, and interestingly, following multivariate analysis, this factor reached statistical significance (OR: 5.21; 95% CI: 1.01–26.77; *p* = 0.048). In addition, Stapf et al. observed that female gender had a slightly protective role regarding hemorrhagic presentation. However, their results did not reach statistical significance [23]. While data on this topic is relatively scarce, various factors might be involved such as hormonal and genetic differences.

### Impact of associated aneurysms on rupture time

In our study, the presence of an associated aneurysm did not statistically affect the rupture rate, although there seemed to be a trend towards a lower rate of rupture in cases where an associated aneurysm was present. However, given the small number of cases with an associated aneurysm, this data should be interpreted cautiously. Interestingly, we found that patients with an associated aneurysm presented with ruptured AVMs later in life, having a median time to rupture of 51 years, compared to patients without associated aneurysms, in which case the median time to rupture was 21 years (HR: 0.32; 95% CI: 0.14–0.71; *p* = 0.016). Garzelli et al. observed a similar correlation between associated aneurysms and delayed rupture and hypothesized that they usually form later due to hemodynamic consequences which lead ultimately to rupture [8].

### Factors influencing the outcome

Bleeding status of the AVM significantly impacts the outcome. We observed that unruptured lesions had an overall better outcome (*p* = 0.039), 93% of the cases having a mRS ≤ 2 at discharge. In contrast, in the ruptured AVMs subgroup only 62% of the patients had a similar outcome. Other authors reported that hemorrhagic presentation is associated with less favorable postoperative outcome, although the condition of patients can improve over time [2, 3, 13]. Lawton et al. also stress the fact that patients with ruptured AVMs have a worse clinical presentation, which can mask the outcome, and therefore, the risk of neurological worsening associated with unruptured AVMs following surgical resection should not be underestimated [13].

In our cohort, 40% of the patients also had intraventricular hemorrhage which proved to be statistically associated with a negative prognosis (*p* < 0.001). Hence, 63% of these cases had a poor outcome on mRS, while only 7% of the patients without IVH had a similar outcome. Other studies support these findings and emphasize that the severity and extent of hemorrhage is associated with poor neurological status [21, 28].Moreover, this observation is reinforced by our findings that in the subgroup of patients with ruptured AVMs, IVH remained an important negative prognostic factor (*p* = 0.001).

We observed that the median SM grade was 2 for cerebellar AVMs, and 3 for brainstem AVMs. As reported in other series, pfAVMs tend to have a smaller grade than the supratentorial ones, mainly due to their reduced size [6, 18]. We found that patients with SM grade I or II lesions have a better outcome compared to patients with grade III or IV malformations (*p* = 0.023) and this finding is more evident in surgically treated patients’ subgroup with ruptured pfAVMs (*p* = 0.009), where SM II malformations have a better prognosis than grade III or IV. As reported by Yilmaz et al., for ruptured posterior fossa AVMs, there is a direct correlation between the SM grade and surgical outcome [30]. This can be explained by the characteristics of higher SM grade AVMs such as more complex angioarchitecture and larger size, which affect the initial neurological status and make the management of this cases more challenging.

Deep venous drainage was associated with poor outcome (*p* = 0.021) across our whole cohort. As Pohjola et al. state, there are many explanations for this crucial role of the venous drainage, being the only factor to influence early and late outcome in their cohort. Lesions that drain in deep veins, most likely also have an arterial supply from a deep artery, making them even more difficult to handle during surgery. Additionally, after occluding a deep vein, the outflow reorganization might lead to unwanted consequences in eloquent areas of the posterior fossa (brainstem, cerebellar nuclei) [18]. Compared to arteries, veins also tend to rupture more easily during surgery and are more difficult to coagulate [16]. The impact of the deep venous drainage is also emphasized by the fact that it is the only factor from the original SM scale that is included in the novel grading scale developed for cerebellar AVMs, being associated with a poor outcome [16].

### Treatment considerations

In our subgroup of surgically treated patients, we observed that 90% of unruptured pfAVMs had a favorable outcome (mRS 0–2), while patients with ruptured AVMs had a favorable outcome in only 44% of the cases (*p* = 0.021). Moreover, a poor mRS score at admission was the only factor that remained independently associated with a poor outcome following multivariate analysis in the surgically treated subgroup (OR: 96.14; 95% CI: 5.15–1793.9; *p* = 0.002). Other studies underline that a favorable pretreatment mRS is associated with good outcome [4, 6, 29]. In addition, Yang et al. found a statistically significant correlation between hemorrhagic presentation and worse mRS score before treatment. These findings suggest that an early aggressive treatment after the diagnosis of a unruptured pfAVM (before a potential rupture, or worse, a new episode of hemorrhage in case of ruptured pfAVMs) might increase the chances of a good functional outcome [1, 4, 23].

Data on this topic is controversial, especially after the ARUBA trial [15]. However, given that the ARUBA trial included only 12 patients with pfAVMs, its findings have limited relevance for this subgroup. In our cohort of unruptured pfAVMs (*n* = 14), we observed no statistical difference in outcomes between patients managed surgically and those undergoing other treatment modalities (*p* = 0.714), emphasizing that surgery offers good outcomes as well. Nonetheless, the subgroup of non-surgical patients was small (*n* = 4) and heterogenous regarding treatment modalities.

A more recent study, the TOBAS trial, showed promising results regarding the preventive role of surgery for unruptured AVMs, especially low-grade [5]. It also raised even more concerns regarding the role of preoperative embolization since many of the adverse events that were noted in the surgical cohort were attributable to embolization [5].

Another argument for an early surgical management of unruptured pfAVMs is that we observed no differences regarding the outcome of surgically treated unruptured AVMs based on the SM grading compared to other treatment modalities. By contrast, as discussed earlier, ruptured AVMs had a better outcome following surgery when the SM grade was lower. Nevertheless, this data should be interpreted with caution, as there were fewer high-grade AVMs in the unruptured subgroup.

In our cohort, most AVMs presented with rupture, and 33 were operated. Of these, in 76% of cases, complete resection was achieved. Multiple studies state that complete and preferable single modality occlusion is the main goal to prevent future hemorrhage and maximize results [4, 6, 14, 27], but in some difficult cases, especially in posterior fossa or deep location, multimodal treatment can represent a safe and feasible option [11, 26, 27]. Based on our findings, as well as recommended by other authors [10], the clinical status of the patient should be taken into account when choosing the optimal treatment strategy.

### Limitations

Our study has limitations. First, it is a retrospective study over a long period of time. Because of its design and the fact that it is a single center study, some degree of bias exists. Second, treatment modalities and techniques have evolved during this time, so obliteration rates are higher and possible complications fewer and easier to manage now. Third, a few patients were lost to follow-up and not in all cases angiography was available. However, CTA or MRA were obtained in these cases, imaging studies were not homogenous. Although the substantial size of this series provides statistically significant correlations that concur with previously published studies, the results of certain subgroup analysis should be interpreted with caution. Although there was a significant difference between the patients with and without associated aneurysms regarding the time to rupture, this data should be carefully interpreted since in our series we had a relatively low number of patients with associated aneurysms, and the calculation of time to rupture implies that all AVMs are congenital lesions.

## Conclusions

Posterior fossa AVM are challenging lesions, not only due to their location, but also because of their tendency to present with hemorrhage. Any bleeding in such a narrow space like the posterior fossa might have hazardous consequences and understanding pfAVMs natural history is of tremendous importance. Unruptured lesions have a better outcome in comparison to those presenting with hemorrhage. The risk of hemorrhagic presentation is significantly higher in patients ≤ 30 years old. Poor mRS (3–5) at admission is the only factor independently associated with poor outcome in the surgically treated patients. Treatment recommendations are clear for ruptured AVMs. Regarding unruptured lesions located in the posterior fossa, after careful selection, treatment can prevent future hemorrhage and maintain good functional outcome.

## Data Availability

The data analyzed in this study is available upon request.

## Abbreviations

AVM: Arterio-venous malformation
CPA: Cerebello-pontine angle
CTA: Computed tomography angiography
IVH: Intraventricular hemorrhage
GCS: Glasgow Coma Scale
MRA: Magnetic resonance angiography
mRS: Modified Rankin scale
pfAVM: Posterior fossa arterio-venous malformation
SM: Spetzler-Martin grade
